# Cisplatin-induced oxidative stress, apoptosis, and pro-inflammatory responses in chondrocytes through modulating LOX-1

**DOI:** 10.1186/s13018-025-05602-9

**Published:** 2025-02-26

**Authors:** Chin-Hsien Wu, Wan-Ching Chou, I.-Ming Jou, Yuan-Kun Tu, Ching-Hou Ma, Kun-Ling Tsai

**Affiliations:** 1 Department of Orthopedics, E-Da Hospital, I-Shou University, Kaohsiung City, Taiwan, ROC; 2 Department of Physical Therapy, College of Medicine, National Cheng Kung University, Tainan, Taiwan, ROC; 3 School of Medicine, I-Shou University, Kaohsiung City, Taiwan, ROC; 4 Institute of Allied Health Sciences, College of Medicine, National Cheng Kung University, Tainan, Taiwan, ROC

**Keywords:** Cisplatin, LOX-1, N-acetyl cysteine, Oxidation stress, Apoptosis

## Abstract

Cisplatin is a potent and efficacious anticancer medication. In pediatric cancer, the height of the growth plate’s proliferating layer is known to be reduced by cisplatin, but researchers have not yet determined the specific mechanism behind this phenomenon. Lectin-like oxidized low-density lipoprotein receptor-1 is known to be involved in the development of osteoarthritis and atherosclerosis. The equilibrium of cartilage is regulated by LOX-1, but the function of LOX-1 in cisplatin-induced chondrocyte impairment remains unknown. Positive regulation of LOX-1 leads to increased cellular oxidative stress and cell damage. Research has shown that blocking of LOX-1 can reduce the chondrocyte damage and oxidative stress in cells induced by oxidized LDL treatment. However, the role of LOX-1 in cisplatin-mediated chondrocyte damage is still unclear. This study found that cisplatin increased ROS concentration and p38, ERK phosphorylation. Cisplatin activated NF-κB in chondrocytes. In addition, LOX-1 small interfering RNA transfection mitigated cisplatin-induced apoptosis in TC28a2 cells. Phosphorylated extracellular signal-regulated kinase and p38 were dose-dependently increased by administration of cisplatin. Silencing LOX-1 or MAPK inhibition reduces cisplatin-caused apoptosis. The findings suggest that cisplatin-induced growth plate dysfunction operates through the LOX-1/p38/NF-κB signaling pathway.

## Introduction

In various cancer cells, programmed cell death can be triggered by certain medications designed to combat cancer [1]. The most common cancers in children, as outlined in a cancer surveillance report, include non-Hodgkin lymphoma, neuroblastoma, central nervous system cancers, and acute lymphocytic leukemia (ALL) [2]. Advancements in cancer treatment have significantly improved the rates of survival among children with cancer, yet concerns persist regarding the potential long-term adverse effects of these treatments. Research has identified various health risks associated with cancer therapies [3]. For instance, the risk of diabetes mellitus is increased in patients receiving radiation directed at the pancreas [4], and chemotherapy may cause gonad toxicity [5], ototoxicity [6], or nephrotoxicity [7]. Studies also reported that chemotherapeutic agents can stunt the growth of growth plate chondrocytes because these agents affect the cells’ proliferative capacity. Notably, cisplatin, a platinum-containing drug, was shown in one study to inhibit chondrocyte proliferation and disrupt the proliferating layer of the growth plate, thus affecting bone growth. Moreover, cisplatin is associated with hypomagnesemia, which can impede bone growth by reducing the activity of osteoblasts and osteoclasts [8]. Despite evidence of cisplatin’s effects of bone growth impairment and growth plate dysfunction, the precise molecular mechanisms underlying these effects remain unclear. Cisplatin is a crucial component of chemotherapy regimens for pediatric cancers that include germ cell tumors, retinoblastoma, brain tumors, hepatoblastoma, osteosarcoma, and neuroblastoma [9, 10]. Ototoxicity is one of cisplatin’s commonly known side effects and has been demonstrated to lead to sensorineural hearing impairment in both ears [10]. The mechanism of cytotoxicity of cisplatin is that the molecule enters a cell, binds to its DNA, and then inhibits DNA synthesis and the growth of the cell [11]. Medications such as cisplatin, actinomycin D, and doxorubicin have been shown in vitro to directly affect growth plate chondrocytes, resulting in decreased growth and final height of animals [12, 13]. Final height and bone growth can be decreased by antimetabolites, as determined in clinical studies involving multiagent chemotherapy. Renal toxicity is thought to be the cause of cisplatin-induced hypomagnesemia, possibly because the way magnesium is reabsorbed in the kidneys is damaged or prevented [14].

Reports on animal studies have revealed that medications such as doxorubicin, actinomycin D, and cisplatin directly influence growth plate chondrocytes, causing a decline in their growth and eventual animal height reduction [15]. The effects of these medications facilitate the specific binding of oxidized low-density lipoprotein (Ox-LDL) and how this molecule is internalized and degraded [16]. Polymorphonuclear leukocytes were discovered in one study to adhere to LDL receptor 1 (LOX-1)-coated surfaces, and this adherence was found to be impeded by anti-LOX-1 antibodies; the researchers accordingly concluded that LOX-1 may be involved in adhesion mechanisms [17]. Additionally, Ox-LDL–LOX-1 binding was noted to trigger reactive oxygen species (ROS) production in cultured bovine articular chondrocytes. Ox-LDL exposure resulted in heightened intracellular oxidative stress in chondrocytes [18]. The use of cisplatin, which has been demonstrated to be efficacious against various cancers and in preclinical and clinical contexts, is limited due to the resistance of some cancer cells to the drug[19]. Cisplatin induces cell death by promoting the generation of free radicals, particularly ROS [20]. However, cisplatin-induced cell death is not necessarily conventional apoptosis [11]. Depending on the cellular conditions in a patient and the cisplatin dosage, this drug may cause cell death through a flawed apoptotic pathway. In addition to inducing oxidative stress, cisplatin triggers mitogen-activated protein kinase (MAPK) phosphorylation [21].

NF-κB is a transcription factor that modulates gene expression in inflammation, immunity, cell proliferation, and cell survival. Cisplatin treatment promotes oxidative stress and further activates NF-kB signaling. On the other hand, ROS elevation by cisplatin treatment causes cellular damage and, in turn, activates NF-kB, leading to a cycle of inflammation and injury [22]. The biological function of NF-kB and MAPK in chondrocyte injuries is particularly relevant to joint diseases such as osteoarthritis (OA). NF-κB is pivotal in mediating inflammatory events and matrix degradation. Up-regulation of NF-κB leads to matrix metalloproteinases (MMPs) activation, thereby contributing to cartilage destruction [23]. The activation of MAPK pathways in chondrocytes leads to the release of pro-inflammatory cytokines and MMPs, further leading to cartilage degradation and chondrocyte dysfunction [24].

Studies have indicated that ROS governs LOX-1 activation. This LOX-1 overexpression causes a rise in ROS in chondrocytes, which initiates a feedback loop that prolongs oxidative stress and death. Thus, the interplay between LOX-1 activation, activation of oxidative stress, and MAPK activation by cisplatin may influence apoptosis in chondrocytes. To mitigate the adverse effects of cisplatin on bone growth and cartilage integrity in pediatric cancer patients, it may be possible to understand better the specific function of LOX-1 in cisplatin-induced chondrocyte death. We hypothesized that cisplatin increased LOX-1 expression by enhancing oxidative stress and MAPK/NF-κB expression.

## Materials and methods

### Cell culture

For this study, we obtained TC28a2 human chondrocytes from Millipore Sigma (St. Louis, MO, USA). Dulbecco’s modified Eagle’s medium (DMEM)/F-12 medium containing penicillin–streptomycin as well as 10% fetal bovine serum (FBS) was employed to culture the aforementioned cells. Millipore Sigma was also the source of the reagents—including FBS, trypsin–ethylenediaminetetraacetic acid, LY3214996, SB203580, PDTC, Lamin B1, 2’,7’-dichlorodihydrofluorescein diacetate (DCF-DA), NAC, and cisplatin—used in the present study and also specific antibodies against phospho-p38, p38, β-actin, Bax, Bcl-2, cytochrome c, ERK, and LOX-1. Moreover, Cell Signaling Technology (Danvers, MA, USA) was the source of horseradish peroxidase (HRP)-conjugated secondary antibodies used in the study. To stimulate chondrocytes with cisplatin, chondrocytes were seeded in 6-cm-diameter dishes containing complete culture medium (DMEM/F-12 supplemented with 10% FBS), with the seeding density being 1 × 10^6^. Subsequently, the dishes were placed in a CO_2_ incubator from Thermo Scientific. Once incubation had been conducted for 36 h, 1 × PBS was used to wash the cells, after which they were treated with 10 μM cisplatin for an extra 24 h in complex culture medium. Some cells were pretreated with NAC; in this case, they were pretreated with 5 μM NAC for 2 h before they were exposed to cisplatin.

### Western blot assay

The cells underwent lysis, which was performed using radioimmunoprecipitation lysis buffer that was supplemented with the following: NaF and Na_3_VO_4_ (phosphatase inhibitors both administered at 1 mM) and leupeptin (administered at 10 g/mL), pepstatin A (administered at 10 g/mL), aprotinin (administered at 10 g/mL), and 4-(2-aminoethyl) benzenesulfonyl fluoride (protease inhibitors administered at 1 mM). Sigma-Aldrich was the source of both types of inhibitor. Subsequently, we combined electrophoresis sample buffer from Bio-Rad Laboratories with equal quantities of protein and then boiled the mixture for 5 min; the mixture was subsequently loaded onto sodium dodecyl sulfate–polyacrylamide gel electrophoresis (SDS-PAGE) gel. SDS-PAGE was performed to execute protein separation, and afterwards, the separated proteins were moved onto Millipore nitrocellulose membranes, which were next incubated with primary antibodies from Cell Signaling Technology (Beverly, MA, USA) and then HRP-conjugated secondary antibodies from the same supplier. The primary antibodies targeted phosphorylated (p-)p38, p-ERK, NF-kBp65, ikBa, LOX-1, Bcl-2, Bax, cytochrome-c, β-actin, and LaminB1.

### ROS assay

A DCFH-DA staining assay kit (Beyotime Biotechnology, Shanghai, China) was employed to determine the levels of ROS in cells. Chondrocytes plated in dishes were exposed to basic culture medium containing DCFH-DA (in a ratio of 1000:1) for 30 min by following established experimental protocols. Subsequently, a fluorescence microscope was utilized to capture random fields of the samples; this instrument was supplied by Olympus Inc. (Tokyo, Japan). We obtained relative fluorescence unit (RFU) values as follows: assay well RFU values − blank well RFU values. A multifunction fluorescence microplate reader was used to perform detection at 490/520 nm.

### TUNEL assay

An in situ terminal dinucleotidyl transferase (TdT)-mediated dUTP nick end-labeling (TUNEL) assay was conducted, with an in situ apoptosis detection kit being used to execute chondrocyte apoptosis detection. The cells under investigation were fixed in acetone and then treated for 5 min with permeabilization buffer at 4 °C. Subsequently, 0.3% H_2_O_2_/methanol was employed to perform blocking, which was followed by 90-min incubation in 50 μL of TdT-labeling solution at 37 °C. Next, the cells were subjected to a washing process, after which they were incubated for 30 min with biotinylated anti-FITC antibody at room temperature. We used 0.025% DAB/PBS to visualize apoptotic cells; counterstaining was conducted using methyl green. For four randomly chosen fields in each slide, we calculated what percentage of the cells were TUNEL-positive; the magnification in this procedure was 200 × .

### Statistical analysis

We present data herein as means ± standard deviations (SDs). We executed a one-way analysis of variance (ANOVA) followed by Tukey’s post hoc test for statistical analyses in cells with variant treatments. Graphs were constructed using OriginLab2020. We considered *p* < 0.05 to indicate statistical significance.

## Results

### Cisplatin increases ROS production in TC28a2 cells

**Fig. 1:**
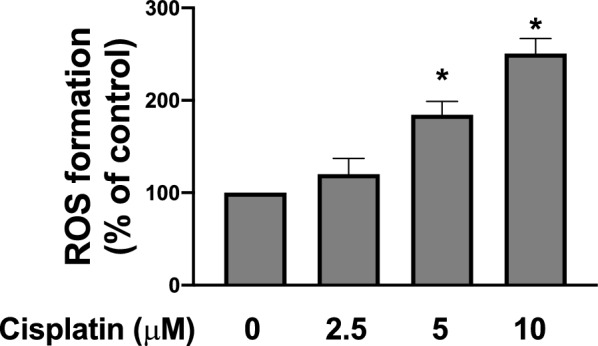
Cisplatin increased ROS concentration in TC28a2 cells. We measured ROS levels by using DCFH-DA after treatment with cisplatin administered at various gradient concentrations. Data are expressed as percentages of the level for the PBS-treated control group and as the mean ± SD (n = 4). *p < 0.05 compared with control

Our Fig. 1 shows that after treating TC28a2 cells with cisplatin (2.5–10 µM) for 24 h. The intracellular ROS increased in a dosage-dependent manner. The dosage of 5 µM and 10 µM was significantly higher than the control group.

### Cisplatin increased p-ERK and p38 through increasing oxidative stress in TC28a2 cells

**Fig. 2:**
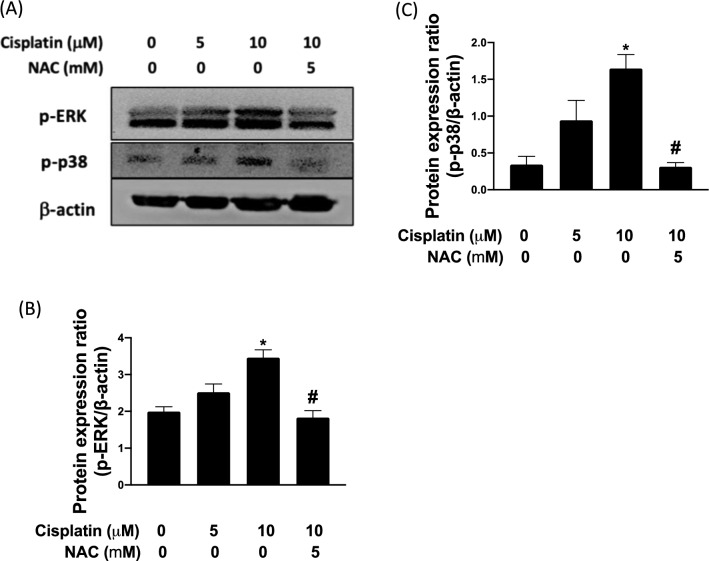
Cisplatin-induced ERK and p38 phosphorylation by increased ROS in TC28a2 cells. **A** Expression of total and phosphorylated ERK and p38 was determined through Western blotting. **B** Protein expression ratio of p-ERK. Expression of this protein was upregulated after cisplatin treatment but downregulated after NAC treatment. **C** Protein expression ratio of p-p38. Expression of this protein was upregulated after cisplatin treatment but downregulated after NAC treatment. The protein expression of p-ERK increased after treatment with cisplatin administered at various gradient concentrations. The protein expression of p-p38 increased after treatment with cisplatin administered at various gradient concentrations. Both p-ERK and p-p38 expression levels were significantly lower than in the group treated with cisplatin only. *p < 0.05 comparison with control; and #*p* < 0.05: comparison with cisplatin group, respectively

We executed Western blotting to assess the p-ERK and p-p38 levels (Fig. 2A). The results indicated a concentration-dependent increase in the p-ERK:p-p38 ratio, as shown in Fig. 2B, C. Upon treatment with antioxidant (NAC), the cells exhibited lower p-ERK and p-p38 expression, suggesting that cisplatin activated the ERK/p38 MAPK pathway through increasing oxidative stress.

### Cisplatin-induced inflammatory responses attenuated by modulation of ERK and p38 signaling pathway

**Fig. 3:**
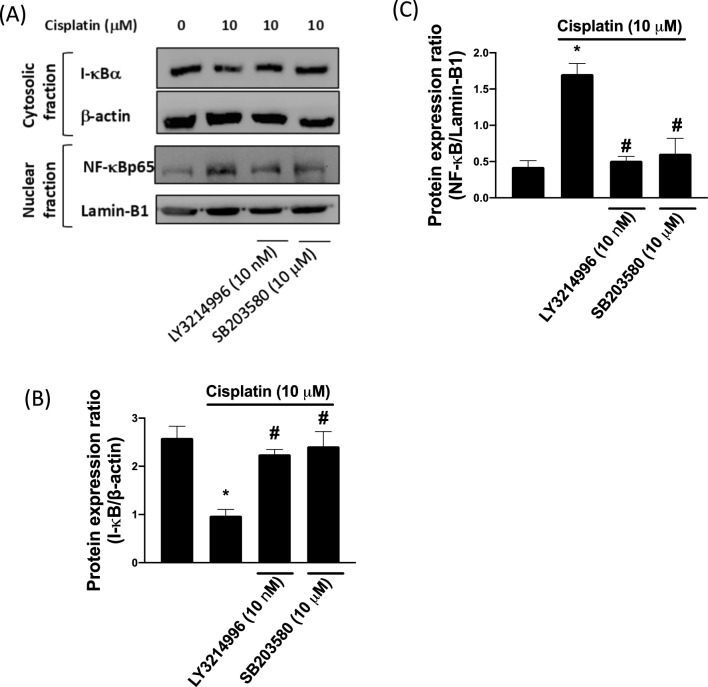
Cisplatin enhanced the NF-κBp65 translocation. **A** Expression of cytosolic I-κBα and nuclear NF-κBp65 was evaluated through Western blotting. **B** Protein expression ratio of I-κBα. Expression of this protein was upregulated after treatment with p38 inhibitor SB203580 and ERK inhibitor LY3214996. **C** Protein expression ratio of NF-κBp65, Expression of this protein was downregulated after treatment with p38 inhibitor SB203580 and ERK inhibitor LY3214996. The differences between the cisplatin-treated group and the ERK inhibitor and p38 inhibitor groups were significant. *p < 0.05 comparison with control; and #*p* < 0.05: comparison with cisplatin group, respectively

To further investigate the activation of the NF-κB signaling pathway after cisplatin treatment, we performed Western blotting to determine the expression levels of I-κBα and nuclear translocation of NF-κBp65 proteins. Figure 3A reveals that the phosphorylation level of NF-κBp65 was raised and I-κBα was decreased in the chondrocytes treated with cisplatin. Treatment with ERK and p38 inhibitor (LY3214996 and SB203580) resulted in a decreased level of NF-κBp65 and an increased level of I-κBα. From these findings, we suggest that cisplatin induces chondrocyte NF-κB activation through modulating ERK/p38 MAPK signaling pathway.

### Cisplatin-induced LOX-1 up-regulation

**Fig. 4:**
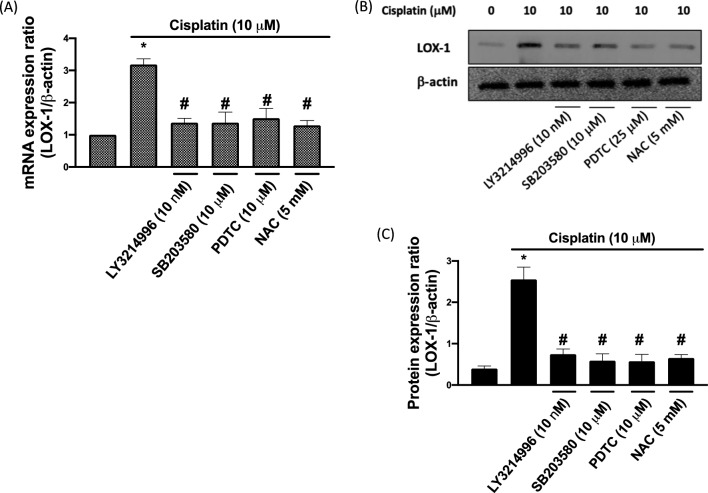
Cisplatin increased LOX-1 upregulation. **A** LOX-1 mRNA expression ratio. This ratio was increased after cisplatin treatment but decreased after three inhibitor treatments and NAC treatment. **B** LOX-1 protein expression was analyzed using Western blotting. **C** Cisplatin-enhanced LOX-1 expression through the ERK–p38–NFkbp65-related pathway. LOX-1 expression was downregulated after three inhibitor treatments and NAC treatment. Significant differences were obtained when comparing three inhibitor treatments and NAC treatment with cisplatin treatment alone. *p < 0.05 comparison with control; and #*p* < 0.05: comparison with cisplatin group, respectively

After their exposure to cisplatin, the TC28a2 cells were treated with ERK, p38, NF-κB inhibitor (LY3214996, SB203580, and PDTC), and NAC. We found that LOX-1 mRNA (Fig. 4A) and protein (Fig. 4B, C) were significantly higher than the control cells in the cisplatin-treated cells. In addition, the cisplatin-caused LOX-1 up-regulation expression was significantly reduced by ERK, p38, NF-κB inhibitor, and NAC, indicating that cisplatin-increased LOX-1 up-regulating by regulation ROS-mediated ERK/p38 MAPK/ NF-κB signaling.

### Cisplatin induces apoptosis in TC28a2 cells through the cisplatin-treated ROS-mediated LOX-1 pathway

**Fig. 5:**
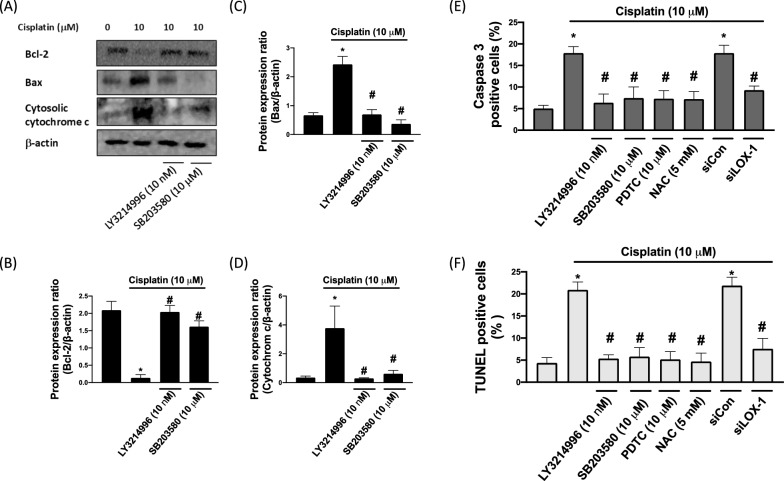
NAC mitigates cisplatin-induced apoptosis through LOX-1 and MAPK mechanisms in chondrocytes. **A** Apoptosis markers—cytosolic cytochrome c, Bax, and Bcl-2—were evaluated through Western blotting. **B**–**D** Calculated protein expression ratios (mean + SD). **E** Caspase-3 and **F** TUNEL expression in the cisplatin-stimulated chondrocytes treated with the ERK inhibitor, p38 inhibitor SB203580, NF-κb inhibitor, NAC and LOX-1 siRNA. *p < 0.05 comparison with control; and #*p* < 0.05: comparison with cisplatin group, respectively

We performed Western blotting to determine the levels of cytosolic cytochrome c, Bax, and Bcl-2 expression that affect cisplatin-induced chondrocyte apoptosis (Fig. 5A–D). In the chondrocytes, cisplatin was found to significantly decrease and to significantly increase the protein level of Bcl-2 and the protein levels of Bax and cytosolic cytochrome c, respectively. Furthermore, after treatment of TC28a2 cells with ERK and p-38 inhibitors (LY3214996 and SB203580), the protein level of cytosolic cytochrome c and that of Bax were significantly lower compared to cisplatin-treated cells. In order to further study the cisplatin induces apoptosis in TC28a2, we conducted caspase 3 activity (Fig. 5E) and TUNEL (Fig. 5E) assay. We reveled that LY3214996, SB203580, and PDTC inhibitor mitigated cisplatin-induced apoptosis. The NAC and LOX-1 silencing also prevented cisplatin-caused apoptosis in TC28a2.

## Discussion

A growing number of studies are reporting that in children with cancer, many cancer treatments and comedications can induce apoptosis in chondrocytes and dysfunction in the growth plate, potentially resulting in growth impairment [25, 26]. One study revealed that chondrocyte apoptosis and osteoclast migration and formation could be induced by high-dose methotrexate or dexamethasone; the mechanism underlying these effects is likely to be these drugs’ stimulation of stromal cell-derived factor 1 (SDF-1) [25]. In other research, radiation-induced ROS triggered type II collagen loss and cellular senescence in chondrocytes through activation of the p38 kinases, leading to the reduction of SIRT1 [27]. We discovered that cisplatin increases oxidative stress and triggers inflammation and apoptosis by activating LOX-1 in chondrocytes.

Cisplatin, known for its efficacy in treating various pediatric malignancies through chemotherapy, has been shown to impede the progression of the cell cycle, resulting in growth plate chondrocytes growing more slowly in vitro [28, 29]. The epiphyseal growth plate contains rapidly dividing chondrocytes that organize into longitudinal columns within the proliferative zone before they go on to grow in the hypertrophic zone. A study conducted on Wistar rats revealed that their trabecular volume and the height of their proliferating layer were reduced when cisplatin was administered [29]. Evidence thus suggests that cisplatin treatment leads to a slowdown in longitudinal growth, but it remains unclear precisely how cisplatin exerts chondrocyte damage. We thus investigated how cisplatin affects chondrocytes by inducing ROS and its signaling pathways.

Chondrocyte dedifferentiation and LOX-1 expression are regulated by various pathways—including MAPKs (p38 and ERK) and phosphoinositide 3-kinase (PI3K)/Akt, along with c-Jun N-terminal kinase—through the effects of these pathways on ROS production [30, 31]. While the traditional focus of research has been on macrophages and endothelial cells as targets of Ox-LDL. A study indicated an increase in LOX-1 expression in response to Ox-LDL, LOX-1’s ligand, and the pro-inflammatory cytokine interleukin (IL)−1β found in osteoarthritic cartilage [32]. Although ROS have been suggested by some researchers to induce LOX-1 expression and inhibit proliferation [18, 32], the mechanisms through which these effects are exerted remain unclear. Our executed study explored how the ROS generated due to cisplatin exposure affects LOX-1 expression in chondrocytes. The findings reported herein indicate that the ROS generated following cisplatin exposure modulates ERK/p38/MAPK signaling, with this signaling resulting in more ROS and apoptosis and inflammation in TC28a2 cells.

The p38 MAPK pathway is established to be involved in generating various matrix metalloproteinases (MMPs) and inflammatory mediators in chondrocytes, such as IL-8, MMP-1, MMP-13, and cyclooxygenase (Cox)−2 [33, 34]. Additionally, oxidative stress and LOX-1 expression were shown to be induced in chondrocytes by p38 MAPK phosphorylation [35]. Experimental cell stressors—such as hyperosmolar conditions, UV irradiation, and heat shock—and inflammatory stimuli—such as TIR and TNFR family ligands—strongly activate p38 MAPKs [36]. These kinases are often referred to as stress-activated kinases. By contrast, a broad spectrum of stimuli—such as microbial products, cytokines, and growth factors—triggers a response in ERKs 1 and 2 [37, 38].

At LOX-1 in endothelial cells, the binding of Ox-LDL triggers ROS production. Consequently, the mechanisms of chondrocyte death may involve ROS production mediated by LOX-1 [18, 32]. NF-κBp65 is a transcription factor sensitive to oxidative stress. In this study, cisplatin-induced LOX-1 overexpression was rapidly decreased through treatment with the inhibitors LY3214996, SB203580, and PDTC, and LOX-1 expression was attenuated by NAC. From these findings, we hypothesize that the ERK/p38MAPK pathway controls LOX-1 regulation and has a significant part to play in the apoptosis of chondrocytes. Treatment with ox-LDL triggers apoptosis in human coronary artery endothelial cells, with this process being facilitated by LOX-1[39]. Ox-LDL was discovered to trigger caspase-3 activation through LOX-1. This activation pathway is associated with reduced expression of Bcl-2 and c-IAP-1 and Smac and cytochrome c release, resulting in caspase-9 activation [40].

We have several limitations in this study. First, we did not conduct an animal study to confirm our findings. Second, although immunofluorescence staining is a commonly used method for visualizing protein localization and expression, it was not performed in this study. However, the results obtained from our current experimental approach sufficiently support our conclusions. Future studies may incorporate immunofluorescence to confirm these findings further.

The present study demonstrated the use of cisplatin in cancer treatment to potentially cause cell damage, including ROS production, a pro-inflammatory response, and apoptosis. To investigate the relationship and signaling pathway, NAC, an antioxidant agent, was used to examine ROS levels, and LOX-1 expression was found to be enhanced. The pro-inflammatory factors ERK and p38 were also discovered to be involved in the cisplatin-induced signaling pathway. Cisplatin augmented NF-κBp65 expression and concurrently attenuated I-κBα expression; however, suppressing ERK and p38 resulted in opposite responses. Cisplatin-induced apoptosis by activating caspases and induced apoptosis of TC28a2 cells via LOX-1/ROS/p38/NF-κB signaling pathways. We also determined that LOX-1 has a crucial role at the beginning of the signaling pathway that engenders cisplatin-induced apoptosis, and we discovered upregulation of Bax, cytochrome c, and caspase-3. Therefore, suppression or inhibition of LOX-1 represents a promising therapeutic strategy for mitigating inflammation-induced damage to cartilage.

## Data Availability

The datasets generated and analyzed in the current study are available from the corresponding author on reasonable request.
